# Therapeutic potential of zinc ^64^Zn aspartate for obesity management: impact on oxidative stress, lipid metabolism, pancreas and liver in high-calorie diet model

**DOI:** 10.3389/fphar.2025.1543166

**Published:** 2025-05-16

**Authors:** Max Temnik, Sergey Gurin, Alexandr Balakin, Roman Byshovets, Olesia Kalmukova, Tetiana Vovk, Tetiana Halenova, Nataliia Raksha, Tetyana Falalyeyeva, Olexiy Savchuk

**Affiliations:** ^1^ Physical Chemistry, Vector Vitale, North Miami Beach, Florida, United States; ^2^ Department of Internal Diseases, Bogomolets National Medical University, Kyiv, Ukraine; ^3^ Educational and Scientific Centre “Institute of Biology and Medicine”, Taras Shevchenko National University of Kyiv, Kyiv, Ukraine

**Keywords:** prooxidant-antioxidant balance, glucose, insulin, fibrosis, NAFLD, 64 Zn-asp, oxidative stress, ROS

## Abstract

Zinc is a critical micronutrient that plays multifaceted roles in oxidative stress management, lipid metabolism, pancreatic function, and liver health, which are all closely interconnected with obesity. Maintaining adequate zinc levels is essential for overall metabolic health and proper functioning of these vital systems. The investigational new drug complex of zinc-64 aspartate (KLS-1 or ^64^Zn-aspartate) was evaluated in this study as a pharmaceutical agent targeting oxidative stress and lipid metabolism using rodent model of obesity. KLS-1 is the isotopically modified zinc aspartate in which stable (non-radioactive) ^64^Zn atoms were enriched to exceed 99% atomic fraction of total zinc, as compared to natural isotopic ratio of 64Zn of 48.6%. In this paper, we discuss our findings and the effects rendered by KLS-1 on lipid metabolism, pancreas and liver function. This study was conducted on outbred rats, which were divided into four experimental groups: 1) the control group consuming standard food (3.81 kcal/g), 2) the obese group consuming a high-calorie diet (5.35 kcal/g), 3) the obese group consuming a high-calorie diet (5.35 kcal/g) treated with intragastric administration of ^64^Zn- aspartate at a dose of 4.5 mg per animal during 6 weeks (the *obese rats*), 4) the group consuming standard food diet (3.81 kcal/g) with ^64^Zn- aspartate form administration. The obese rats treated with ^64^Zn-64 stable isotope demonstrated decreased area of the hepatocytes, insulin and glucose levels in serum; increased catalase and superoxide dismutase activity, and area of pancreatic islets in comparison with the obese group. The study shows that ^64^Zn-aspartate is effective as a therapeutic agent for obesity management, significantly reducing body mass, improving histopathological changes in the pancreas and liver and normalizing oxidative stress in high-calorie diet animal models. These findings suggest that ^64^Zn- aspartate may be a promising monotherapy or adjunct treatment for obesity, offering benefits in weight reduction, organ protection, and antioxidant balance.

## 1 Introduction

Zinc is a critical trace element essential for human health throughout the entire lifespan, from fetal development to old age. It is classified among the indispensable trace elements and essential micronutrients, alongside iron, iodine, copper, selenium, and manganese, among others (Rolić et al., 2024).

Zinc plays a fundamental role in maintaining the metabolic equilibrium of the human body. It is involved in the function of over 200 enzymes, either as a structural component or as a regulator of their activity, spanning all enzyme classes (Maret, 2013). These include transferases (e.g., RNA and DNA polymerases, reverse transcriptase, thymidine kinase, nucleotidyl transferase, carboxypeptidase, and other peptidases), hydrolases (e.g., alkaline phosphatase, 5-nucleotidase, aminopeptidase), lyases (e.g., aldolase, carbonic anhydrase), oxidoreductases (e.g., alcohol dehydrogenase, superoxide dismutase), as well as ligases and isomerases (Pasquini et al., 2022; Hou et al., 2021; Zheng et al., 2022; Clemens, 2022; Hübner and Haase, 2021; Liao et al., 2023). The absence of zinc renders the metabolism of proteins, fats, and carbohydrates impossible.

The metabolic and structural importance of zinc is underscored by its extensive biological activity. Zinc is crucial for processes such as cell division and differentiation (including growth, tissue regeneration, and spermatogenesis) (Garner et al., 2021). It plays an active role in nucleic acid metabolism and protein synthesis (Sun et al., 2023). Furthermore, zinc is vital for the metabolism of polyunsaturated fatty acids and the conversion of prostaglandins (Stawarska et al., 2021). It exhibits significant lipotropic activity and possesses hepatoprotective properties (Wei et al., 2018; Palladini et al., 2022). Zinc deficiency can negatively impact erythropoiesis and hemoglobin synthesis (Chasapis et al., 2020).

Zinc plays a critical role in the metabolism and function of various endocrine glands, including the pituitary, adrenal glands, pancreas, prostate, and testes (Baltaci et al., 2019). It is present within the cells of the anterior pituitary gland, where it participates in metabolic processes and modulates the activity of hypophysiotropic hormones (Nishiuchi et al., 2018). Zinc is instrumental in appetite control and glucose metabolism, which may contribute to its potential benefits in managing obesity (de Vargas et al., 2023). Zinc also extends and enhances the action of adrenocorticotropic hormone (ACTH), amplifies the effects of gonadotropins and growth hormone, and is crucial in the synthesis and biological activity of insulin (Bjørklund et al., 2020). Additionally, zinc acts as both a synergist and antagonist in the absorption and metabolism of various trace elements and vitamins, such as iron, copper, magnesium, and vitamins A, E, and folic acid (Mehri, 2020).

Zinc is thus integral to numerous essential physiological processes in the human body. Despite its importance, the full extent of zinc’s functions remains incompletely understood, with many mechanisms of its action still under investigation. However, existing experimental and clinical studies suggest that zinc is a key element in the body, and its deficiency is linked to the onset and progression of several prevalent non-communicable diseases (Prasad, 2020). Zinc deficiency has been described as “the most prevalent malnutrition in the world” (Brewer and Kaur, 2013). The situation is even more concerning in developing nations. According to World Health Organization (WHO) estimates, approximately 31% of the global population is affected by zinc deficiency, with prevalence rates ranging from 4% to 73% depending on the country. Multiple studies have demonstrated that blood zinc levels were significantly decreased in obese patients (Olechnowicz et al., 2018). A meta-analysis of 23 observational studies found that individuals with obesity had lower concentrations of serum zinc than individuals without obesity (Abdollahi et al., 2020a). Zinc deficiency weakens various cellular functions required for zinc metabolism, including disrupting zinc transport systems, impairing energy metabolism, affecting protein synthesis and function, fading antioxidant defenses, and compromising immune function. These effects create a self-exacerbating cycle where zinc deficiency further impairs the cellular mechanisms needed for proper zinc utilization and homeostasis.

Zinc has antioxidant and anti-inflammatory effects that may help reduce oxidative stress and inflammation linked to obesity, potentially lowering fat accumulation and improving metabolic health (Franco and Canzoniero, 2024a; Banaszak et al., 2021). It also supports adipose tissue function and may aid in managing obesity-related conditions like diabetes and metabolic syndrome (Khorsandi et al., 2019; Banaszak et al., 2021). In metabolic syndrome patients, zinc supplementation improved blood sugar control, lipid levels, and inflammation (Ding et al., 2022).

Plasma zinc concentration, a common indicator of deficiency, is unreliable due to influences like diet, age, sex, medication, and illness (World Health Organization). Zinc deficiency is a risk factor for metabolic diseases such as NAFLD, obesity, and type 2 diabetes, and may result from thyroid or liver disorders, poor absorption, or low dietary intake (Hussain et al., 2022). Needs increase during pregnancy, stress, dialysis, or high physical activity, and deficiency risk rises with certain medications and excessive alcohol use (Stiles et al., 2024).

Zinc is crucial for many metabolic processes, and its deficiency disrupts zinc transport, energy metabolism, protein function, antioxidant defenses, and immunity, creating a cycle that worsens zinc imbalance and cellular dysfunction. Zinc deficiency is linked to obesity, insulin resistance, type 2 diabetes, hypertension, atherosclerosis, and heart disease, worsening disease progression, especially in genetically predisposed individuals (Soheilipour et al., 2021).

The global rise in obesity poses serious medical, social, and economic challenges, affecting all demographics regardless of age, gender, or status (Anekwe et al., 2020; Tak and Lee, 2021). Current obesity treatments often cause side effects and only modest weight loss, underscoring the need for safer, more effective therapies with both preventive and curative potential (Melson et al., 2024).

Recent research also highlights a role for zinc isotope fractionation in human physiology. It is influenced by factors like zinc binding to dietary phytates, affecting absorption and favoring lighter isotopes (Jaouen et al., 2019; Jaouen et al., 2016). However, it remains unclear whether standard dietary supplements can correct these isotopic imbalances.

Zinc is an essential trace element involved in numerous physiological processes, primarily through its role as a cofactor for over 300 enzymes related to protein synthesis, DNA replication, and cellular metabolism (Costa et al., 2023). Due to its limited storage in the body, zinc homeostasis depends on a tightly regulated balance of dietary intake, absorption, distribution, cellular uptake, and excretion, mediated by specific zinc transporters (ZIP and ZnT families) and zinc-binding proteins such as metallothionein (Singh et al., 2023).

Chelated forms of zinc, such as 64Zn-Aspartate, offer improved solubility and bioavailability compared to inorganic zinc salts. Aspartate acts as a ligand, forming a stable complex that facilitates gastrointestinal absorption and may enhance tissue-specific delivery and cellular uptake (Kimura and Kambe, 2016). This chelation may also influence the function of zinc transporters, potentially improving the efficiency of zinc metabolism. Following absorption, zinc is distributed to organs with high metabolic activity, including the liver, muscles, and bones, where it contributes to vital biochemical pathways. Excess zinc is primarily excreted through the gastrointestinal tract and urine, maintaining systemic homeostasis.

Previous studies have demonstrated that zinc may influence obesity-related insulin resistance, hyperleptinemia, inflammation, and the pro-antioxidant balance (Chukwuma et al., 2020).

At this time, there is limited direct information about ^64^zinc excretion in obesity patients specifically. However, normal daily excretion of zinc in urine ranges from 20 to 967 mcg/24 h in healthy adults (Bonaventura et al., 2015) while zinc consumption is insufficient. A study analyzing 60 urine samples from 10 healthy participants found that samples with lower zinc concentrations were systematically enriched in heavy zinc isotopes (FRANCO and CANZONIERO, 2024). This is in line with our hypothesis that healthy cellular functions are maintained with light isotopes of zinc.

Given zinc’s active involvement in metabolic processes that are closely linked to the pathogenesis of obesity, and the finding that healthy humans excrete heavy zinc isotopes, we have hypothesized that an inherently safe pharmaceutical agent containing enriched light atoms of zinc may render a therapeutic effect on oxidative stress and lipid metabolism, as well as on pancreatic and liver function. This study aims to evaluate the effects of ^64^Zn- aspartate on the development of obesity induced by a high-fat diet in experimental animals. The innovative aspect of our study lies in its investigation of the effects of 64Zn-aspartate (KLS-1) on various biomarkers, including serum alkaline phosphatase activity, serum superoxide dismutase activity, catalase activity, serum diene conjugates, Schiff bases, serum TBA-reactive products, and protein oxidative modification. Additionally, this study explores the alterations in pancreatic and liver function in a high-fat diet-induced obesity rat model, providing a comprehensive evaluation of 64Zn-aspartate’s potential therapeutic effects in the context of obesity and metabolic dysfunction.

## 2 Methods and animals

### 2.1 Investigational pharmaceutical agent

**FIGURE 1:**
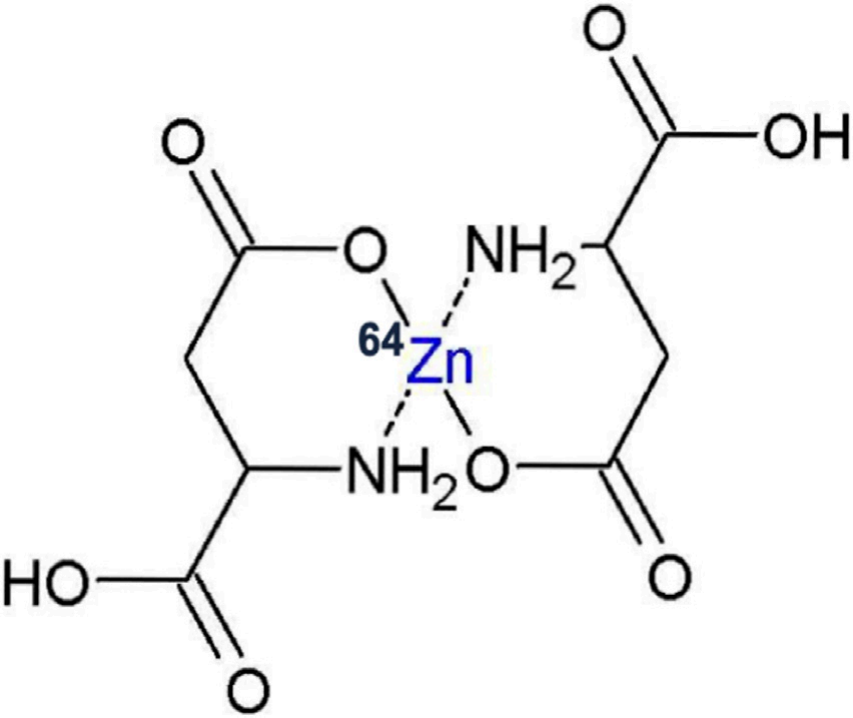
Schematic representation of KLS-1 structure.

^64^Zn-Aspartate (^64^Zn-asp coded “KLS-1”) is a new complex of isotopically modified zinc and L-aspartate, in which the light zinc ^64^Zn isotope is enriched to exceed 99% atomic fraction of total zinc. KLS-1 is a small molecule that is structurally a zinc chelate consisting of two molecules of L-aspartic acid and one non-radioactive (stable) atom of ^64^Zn (Figure 1).

### 2.2 Animal model and experimental design

This study utilized white non-linear rats, which were housed in an accredited vivarium at the Educational and Scientific Center “Institute of Biology and Medicine” Taras Shevchenko National University of Kyiv. The animals were cared for by the Standard Rules on the Arrangement, Equipment, and Maintenance of Experimental Biological Clinics (vivariums) and the study adhered to international standards, including the European Convention for the Protection of Vertebrate Animals used for Experimental and Other Scientific Purposes (Strasbourg, 18/03/1986). The study protocol was approved by the Bioethics Commission of the Educational and Scientific Center “Institute of Biology and Medicine” Taras Shevchenko National University of Kyiv.

The study involved 40 rats with an initial body weight of 200 ± 10 g, maintained on a standard diet before the induction of obesity. To model obesity, the rats were fed a high-calorie diet composed of standard feed (60%), lard (10%), chicken eggs (10%), sucrose (9%), peanuts (5%), dry milk (5%), and sunflower oil (1%). The high-calorie diet was prepared in-house. After 4 weeks on the high-calorie diet, the rats were randomly divided into four groups (10 animals per group):

1. Control Group (C): Rats in the control group were fed a standard diet prepared by the vivarium and had free access to water throughout the experiment.
2. Obesity Group (diet-induced obesity, DIO): Rats in this group continued on the high-calorie diet with free access to water for an additional 6 weeks.
3. Obesity + ^64^Zn-Asp Group (DIO + ^64^Zn): This group also continued on the high-calorie diet and had free access to water. Additionally, these animals were intragastrically administered a solution of ^64^Zn- aspartate at a dose of 4.5 mg per animal in a 2 mL solution every third day.
4. Control + ^64^Zn Group (C + ^64^Zn): Similar to the control group, these rats were fed a standard diet and had free access to water. However, they were also intragastrically administered ^64^Zn- aspartate at a dose of 4.5 mg per animal in a 2 mL solution every third day.

Animals in all groups were weighed once a week following an overnight fast. Daily feed intake was monitored to ensure an accurate assessment of dietary consumption. After a total of 10 weeks on the experimental diets, the animals were sacrificed *via* decapitation.

After the experiment, the Body Mass Index (BMI) was calculated for each animal using the ratio of body weight (g) relative to the square of body length (cm^2^).

### 2.3 Preparation of blood serum

Blood serum was prepared from whole blood samples collected from the experimental animals. To remove fibrinogen-related proteins, the blood was incubated at 37°C for 30 min. After incubation, a blood clot was carefully dislodged from the walls of the tube using a clean, dry glass rod to expedite serum production. The samples were then centrifuged at 2,500 *g* for 15 min. The resulting supernatant (serum) was carefully separated from the blood cells and immediately frozen at −20°C until further analysis.

### 2.4 Determination of glucose concentration in serum

Glucose concentration in the blood of animals, fasted for at least 2 h, was measured using the GLUTOFOT-II glucose meter (LLC “Norma,” Ukraine) following the manufacturer’s instructions. Blood was drawn from the tail vein using a catheter. The glucose concentration was determined *via* the glucose oxidase method. The test strip, containing all necessary reagents, facilitated the formation of a colored complex as a result of the reaction. A drop of whole blood was applied to the strip, incubated at room temperature for 30 s, then washed with distilled water, and analyzed using the glucose meter. Glucose levels were expressed in mmol/L.

### 2.5 Determination of serum alkaline phosphatase activity

Alkaline phosphatase activity in serum was measured spectrophotometrically using a Microlab 300 biochemical analyzer and standard PLIVA-Lachema Diagnostika test kits (Czech Republic). The enzymatic hydrolysis of p-nitrophenyl phosphate by alkaline phosphatase produces p-nitrophenol, which exhibits an intense yellow color in alkaline conditions. The optical density of the samples was measured at 405 nm. Enzyme activity was expressed in relative units.

### 2.6 Determination of serum albumin

Serum albumin levels were quantified spectrophotometrically using a Microlab 300 biochemistry analyzer and standard PLIVA-Lachema Diagnostika test kits (Czech Republic).

### 2.7 Determination of serum superoxide dismutase activity

Superoxide dismutase (SOD) activity was measured based on the enzyme’s ability to inhibit the auto-oxidation of adrenaline. Serum aliquots were added to microplate wells containing 0.2 M bicarbonate buffer, pH 10. The reaction was initiated by adding a 0.1% adrenaline solution to each well. The optical density was measured at 347 nm using a µQuant microplate spectrophotometer (BioTek Instruments, United States) at 4 and 8 min after the addition of adrenaline. SOD activity was expressed in relative units/min/mg (Haftu et al., 2020).

### 2.8 Determination of catalase activity

Catalase activity was assessed using a spectrophotometric method that relies on hydrogen peroxide’s ability to form a stable colored complex with molybdenum salts. The reaction was initiated by adding the test sample to 0.03% hydrogen peroxide. After 10 min, the reaction was halted by the addition of a 4% ammonium molybdate solution. The optical density was measured at 410 nm using a µQuant microplate spectrophotometer (BioTek Instruments, United States). Catalase activity was quantified using a calibration curve and expressed as μmol H_2_O_2_/mg protein x min (Hou et al., 2021).

### 2.9 Determination of diene conjugates and Schiff bases in serum

To assess the levels of diene conjugates and Schiff bases, aliquots containing 0.1–0.5 mg of protein from the test samples were homogenized in a mixture of heptane and isopropyl alcohol (1:1 ratio) using a tight-fitting glass homogenizer for 10 min. The homogenates were then centrifuged at 1,000 *g* for 15 min in tightly sealed test tubes. The supernatant was collected, and distilled water was added to separate the heptane and isopropyl alcohol phases. Schiff base levels were determined in the upper heptane phase by measuring the optical density at an excitation wavelength of 360 nm and an emission wavelength of 420 nm using a spectrophotometer. Schiff base concentrations were expressed in units per mg of protein.

For the determination of diene conjugates, an aliquot of the heptane phase was mixed with 96% ethanol, and the optical density was measured at 233 nm using a spectrophotometer SmartSpec (Bio-Rad, United States). The levels of diene conjugates were calculated using a molar extinction coefficient (2.2 × 10^5^ cm^−1^ × M^−1^) for conjugated dienes formed during the oxidation of polyunsaturated fatty acids and expressed as nmol per mg of protein (Palladini et al., 2022).

### 2.10 Determination of TBA-Active products in serum

The concentration of thiobarbituric acid-reactive substances (TBA-active products) was measured in both serum and adipose tissue homogenates. An aliquot of the test sample was treated with an equal volume of 17% trichloroacetic acid and centrifuged at 1,000 *g* for 15 min (Palladini et al., 2022). The supernatant was then mixed with 0.8% thiobarbituric acid solution and incubated in a boiling water bath for 10 min to allow color development. The optical density was measured at 532 nm using a spectrophotometer SmartSpec (Bio-Rad, United States). The concentration of TBA-active products was calculated using a molar extinction coefficient (1.56 × 10^5^ cm^−1^ × M^−1^) and expressed in nmol per mg of protein.

### 2.11 Determination of oxidative modification of proteins

The oxidative modification of proteins was assessed by measuring protein carbonyls and Schiff bases through their reaction with 2,4-dinitrophenylhydrazine (DNPH), resulting in the formation of 2,4-dinitrophenylhydrazones of neutral and basic nature (Chasapis et al., 2020). An aliquot containing 0.2 mg of protein was mixed with 0.15 M potassium phosphate buffer (pH 7.4). Proteins were precipitated by adding a 20% TCA (Trichloroacetic acid) solution, and the precipitate was centrifuged at 1,000 *g* for 15 min. The precipitate was then treated with 0.1 M DNPH in 2 M HCl and incubated at room temperature for 1 h. After incubation, the precipitate was washed three times with a 1:1 ethanol: ethyl acetate mixture to remove unbound lipids and DNPH, then dried and dissolved in 8 M urea in a boiling water bath for 10 min. The optical density was measured at 356 nm and 370 nm to determine aldehyde and ketone products of oxidative modification, respectively, and was recalculated using appropriate molar extinction coefficients.

### 2.12 Histopathological analysis of pancreatic and liver tissues

At the end of the experiment, liver and pancreas samples (0.5 × 0.5 cm) were immediately placed in a fixative solution (4% paraformaldehyde) at 25°C for 72 h.

Histological paraffin sections, 5 μm thick, were stained with hematoxylin and eosin. For liver fibrosis assessment, Van Gieson’s picro-fuchsin staining method was used. The sections were re-stained with Bömer’s hematoxylin, then with Van Gieson’s picro-fuchsin, and processed similarly to the hematoxylin-eosin staining. Collagen fibers appeared red, hepatocyte nuclei dark brown, and cytoplasm yellow. The quantitative measuring of red collagen fiber (the related area occupied by collagen fiber) was determined as a percentage of the total tissue area. All histological parameters were analyzed using ImageJ software.

### 2.13 Statistical analysis

The distribution of data was assessed with the Shapiro-Wilk normality W-test and variance homogeneity test. One-way ANOVA with Tukey’s *post hoc* multiple comparison tests served for the assessment of significance of the observed changes served for the assessment of the significance of the observed changes. A statistically significant difference was evaluated at p < 0.05 using Origin 8 Pro. Histograms were created using Microsoft Excel 2010 software (Microsoft, United States) and Origin 8 Pro. The obtained results are presented as mean value ± standard error of the mean (SEM).

## 3 Results

### 3.1 Biochemical and anthropometric effects of ^64^Zn aspartate in obesity animal models

To evaluate the impact of ^64^Zn- aspartate form on obesity development induced by high-fat diets, various anthropometric parameters were assessed in obese animal models and in those treated with ^64^Zn aspartate. The experimental data (Table 1) demonstrate that, by the 10th week, the mean body mass index (BMI) of the control animals was 0.60 ± 0.004 g/cm^2^, which falls within the reference range for this age group (Haftu et al., 2020). In contrast, the BMI of animals fed a high-fat diet was 1.14 times higher than that of the control group (0.71 ± 0.002 g/cm^2^). Notably, rats receiving ^64^Zn aspartate treatment exhibited a lower BMI than the obese animals, but slightly higher BMI than the control values (0.65 ± 0.001 g/cm^2^). These findings suggest that ^64^Zn-aspartate exerts a beneficial effect on the metabolic status of the obese animals, providing a foundation for further investigation into the mechanisms underlying its effects on obesity.

**TABLE 1:** Anthropometric values, food intake, and caloric content (M ± SEM, n = 10).

	Experimental groups			
	C	C + zinc	DIO	DIO + zinc
BMI (g/cm^2^)	0.60 ± 0.004	0.59 ± 0.001	0.71 ± 0.002^a^	0.65 ± 0.001^a^ ^,^ ^b^
Weight gain as of the end of the experiment (%)	59 ± 5	59 ± 6	103 ± 11^a^	62 ± 7^b^
Amount of food consumed (g/day)	34 ± 3	32 ± 2	35 ± 1	29 ± 2^b^
Caloric content of food (kJ/day)	525 ± 45	490 ± 32	1,001 ± 54^a^	823 ± 21^a^ ^,^ ^b^

Abbreviations: C, control group; C + Zinc, Control group treated with Zn-64, stable isotope in aspartate form; DIO, diet-induced obesity group; DIO + Zinc, diet-induced obesity group treated with Zn-64 stable isotope in aspartate form.

^a^ The difference is significant compared to the control group of animals (p < 0.05).

^b^ The difference is significant compared to the group of animal models of obesity (p < 0.05).

**FIGURE 2:**
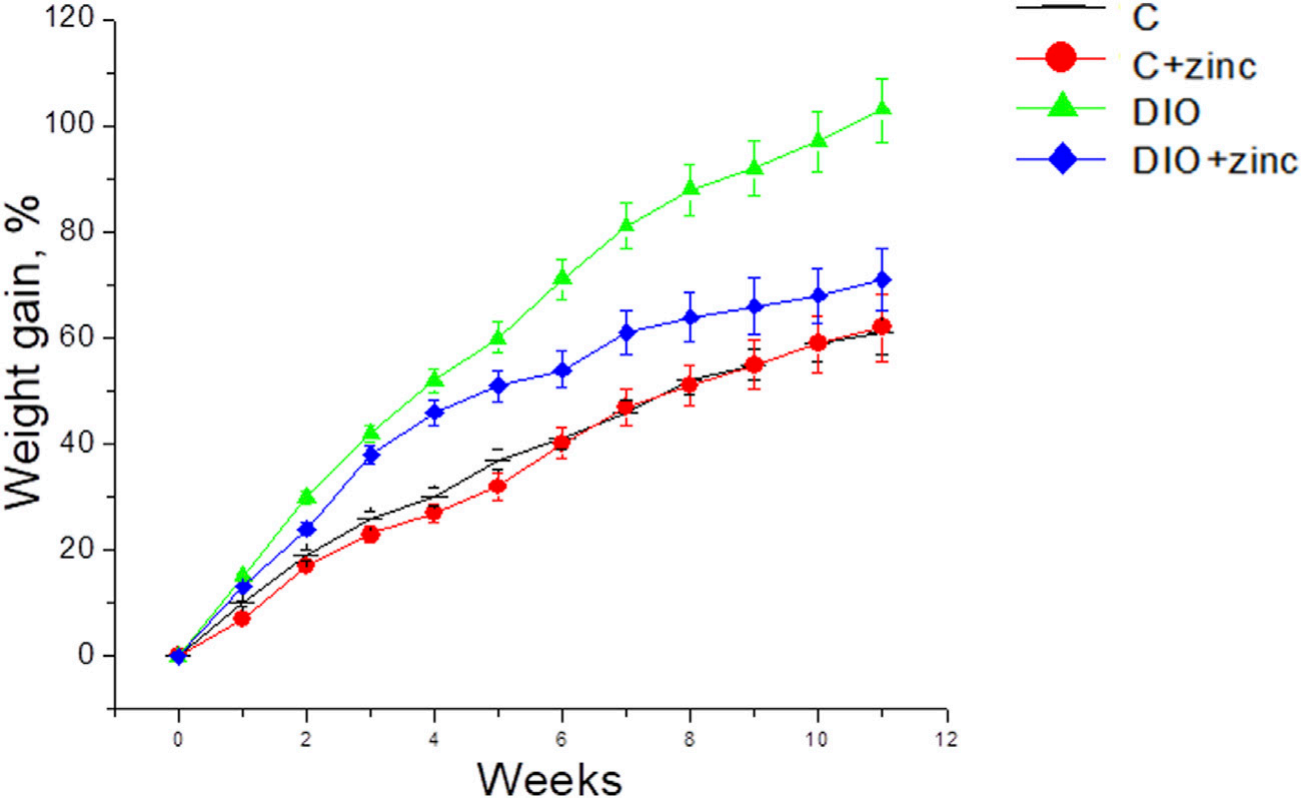
Dynamics of body weight gain in animals in experimental groups (M ± SEM, n = 10).

Given that BMI is calculated based on weight, a reduction in BMI may be directly associated with the lower body weight of animals treated with ^64^Zn- aspartate. Consequently, the effect of ^64^Zn-aspartate on weight and weight gain in obese animal models was further examined. The experimental data (Figure 2) revealed significant differences in the weight gain dynamics among the experimental groups. Animals on a high-fat diet that received ^64^Zn- aspartate gained less weight than those fed the high-fat diet only. The most pronounced difference in weight gain between these groups became apparent from the fourth week of the experiment. By the end of the experiment, animals consuming the high-fat diet experienced a 103% increase in body weight, whereas the animals administered intragastric injections of ^64^Zn- aspartate gained ∼62% weight comparable to the control group.

Obesity develops due to disruptions in the coordinated functions of various neurotransmitter and hormonal systems, leading to impaired control of appetite and regulation of satiety. This dysregulation promotes excessive food intake and is often accompanied by hyperphagia, a condition marked by an abnormally high desire for food, where the energy intake surpasses the body’s energy requirements (Halenova et al., 2018, Halenova et al., 2020).

**FIGURE 3:**
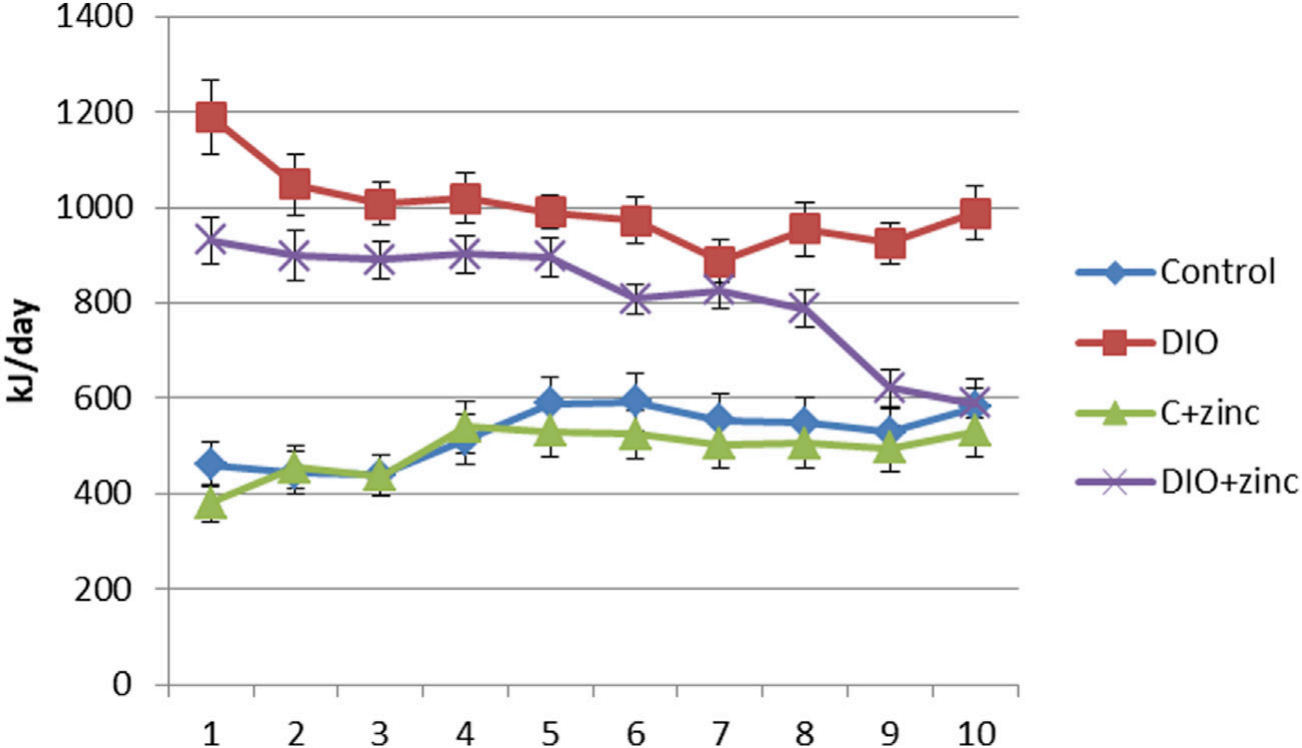
Caloric content of food consumed by animals of experimental groups (M ± SEM. n = 10).

To investigate the mechanisms behind the reduced body weight in 64Zn-aspartate-treated animals, we assessed food intake (Table 1). While both control and DIO groups consumed ∼35 g/day, the DIO diet had nearly twice the caloric content. Notably, animals treated with 64Zn-aspartate consumed less food overall, regardless of diet type. As shown in Figure 3, this reduced intake persisted over 10 weeks, suggesting a potential effect of 64Zn-aspartate on satiety and energy homeostasis. Consequently, treated animals showed reduced weight gain and improved BMI compared to untreated DIO rats. Given zinc’s role in metabolic regulation, early detection and correction of deficiency remain critical, though plasma zinc levels are not always a reliable indicator due to multiple influencing factors (de Moura et al., 2021). Alternative approaches to determining zinc status include measuring the concentrations of zinc-dependent proteins, particularly enzymes such as carbonic anhydrase, superoxide dismutase, lactate dehydrogenase, and alkaline phosphatase, as well as metallothionein and serum retinol-binding protein. One of the earliest markers of zinc deficiency is the reduced activity of serum alkaline phosphatase and carbonic anhydrase (Doulberis et al., 2020). Zinc deficiency can lead to the development of stress ulcers in the gastrointestinal tract, attributed to a decrease in carbonic anhydrase activity in the mucosa (Qu et al., 2020).

To indirectly assess whether obesity is associated with alterations in zinc status, we measured the alkaline phosphatase activity in blood serum of obese animals and those treated with ^64^Zn-aspartate. Our study revealed a significant reduction in alkaline phosphatase activity in animals maintained on a high-fat diet (Table 2). Specifically, enzyme activity in these animals was 1.5 times lower than in the control group. In contrast, animals treated with ^64^Zn-aspartate exhibited higher alkaline phosphatase activity compared to both the DIO group and the control group. These findings indirectly confirm zinc deficiency was developed in obese animal models and suggest that treatment with ^64^Zn- aspartate normalized serum zinc levels.

**TABLE 2:** Biochemical analysis of blood serum of experimental animals (M ± SEM, n = 10).

Groups parameters	C	DIO	DIO + zinc
Alkaline phosphatase activity, CU	74.3 ± 12.1	37.2 ± 15.4^a^	87.6 ± 18.7^b^
Albumin levels, CU	219.2 ± 14.6	168.8 ± 16.8^a^	166.2 ± 15.8^a^
Triglycerides, g/L	2.55 ± 0.20	4.39 ± 0.73^a^	2.79 ± 0.30^b^
Cholesterol, g/L	934.5 ± 73.4	2,224.3 ± 336.6^a^	1,095.5 ± 88.9^b^
Free fatty acids, g/L	0.024 ± 0.005	0.075 ± 0.009^a^	0.032 ± 0.008^b^

Abbreviation: CU, conditional units.

^a^ The difference is significant compared to the control group of animals.

^b^ The difference is significant compared to the group of animal models of obesity.

The gastrointestinal tract plays an important role for maintaining zinc homeostasis throughout the body. Zinc absorbed from the intestine enters the bloodstream, where whole blood typically contains approximately 7–8 mg/L of zinc. Notably, about two-thirds of this zinc is transported by red blood cells. In plasma, around 80% of zinc is bound to albumin, with the remaining 20% bound to β2-macroglobulin and transferrin. Published studies confirm a correlation between zinc levels and the concentration of albumin in blood plasma (Lowe et al., 2024).

Given the role of albumin in zinc transport, we investigated albumin levels in the untreated obese animal models and the obese animals treated with ^64^Zn-aspartate. The experimental data indicates that the pathogenesis of obesity is associated with a decrease in serum albumin levels. The administration of ^64^Zn- aspartate did not significantly affect albumin levels, which remained similar to those in untreated obese animals (Table 2).

As albumin is the primary transport protein for zinc, a decrease in its concentration could disrupt the timely delivery of zinc to organs such as the liver, where the synthesis of key zinc-containing proteins occurs. This finding aligns with the observed decrease in alkaline phosphatase activity noted earlier.

Additionally, ^64^Zn-aspartate form was found to positively influence lipid metabolism. The levels of triglycerides, cholesterol, and free fatty acids in the serum of animals fed a high-fat diet and treated with Zn-64 stable isotope were nearly comparable to those in the control group.

Literature suggests that normal fasting blood glucose levels range from 3.5 to 5.5 mmol/L. An increase in glucose levels to 7.0 mmol/L or higher over time is indicative of hyperglycemia and may predict the development of diabetes mellitus. Our results show that serum glucose levels in the control group and the control group treated with ^64^Zn- aspartate remained within normal reference values (Table 3). The development of obesity led to increase in glucose levels, which were normalized by the administration of ^64^Zn- aspartate.

**TABLE 3:** Serum glucose concentration and insulin level in experimental animals (M ± SEM, n = 10).

Experimental groups	Insulin levels, CU	Glucose levels, mmol/L	HOMA index
C	0.133 ± 0.024	4.4 ± 0.3	0.078 ± 0.001
C + zinc	0.145 ± 0.013	4.7 ± 0.2	0.091 ± 0.001
DIO	0.216 ± 0.035^a^	7.1 ± 0.1^a^	2.044 ± 0.004^a^
DIO + zinc	0.149 ± 0.018^b^	4.9 ± 0.2^b^	0.973 ± 0.005^a^ ^,^ ^b^

Abbreviation: CU, conditional units.

^a^ The difference is significant compared to the control group of animals.

^b^ The difference is significant compared to the group of animal models of obesity.

The glucose-lowering effect of ^64^Zn- aspartate may be attributed to its ability to stimulate the translocation of glucose transporters from intracellular compartments to adipocyte membranes, thereby enhancing intracellular glucose uptake (Campestre et al., 2021). Furthermore, ^64^Zn-aspartate has been shown to increase tyrosine phosphorylation of the insulin receptor β-subunit, improving glucose transport even in the absence of insulin (Grüngreiff et al., 2021). These findings suggest that ^64^Zn-aspartate may act as an inhibitor of tyrosine phosphatase-1B, an enzyme that suppresses insulin signaling (Severo et al., 2020).

Given the observed changes in serum glucose levels, we next examined insulin levels. Serum insulin is a critical parameter for diagnosing insulin resistance and prediabetes. In obesity and metabolic syndrome, hyperinsulinemia is often a compensatory response to decreased sensitivity of peripheral tissues to insulin, leading to excessive insulin production and secretion by pancreatic β-cells. However, in the later stages of type 2 diabetes mellitus, serum insulin levels decrease significantly due to impaired β-cell function, including reduced insulin production, impaired proinsulin processing, and amyloid deposition in the islets. This β-cell dysfunction further exacerbates the progression of diabetes mellitus (Zheng et al., 2022).

Our study revealed elevated serum insulin levels in obese animals, with a normalizing effect observed in the obese rats treated with ^64^Zn-aspartate. Interestingly, the administration of ^64^Zn-aspartate to control group animals also resulted in a slight increase in insulin levels.

Given the critical role of maintaining physiological zinc levels in the body for the synthesis and secretion of insulin, as well as its essential function in pancreatic activity, the effects of ^64^Zn- aspartate on the overall histophysiology of the pancreas were further investigated.

### 3.2 Effect of stable isotope ^64^Zn-64 In the form of aspartate on histopathological changes in the pancreas and liver of DIO animal models

**FIGURE 4:**
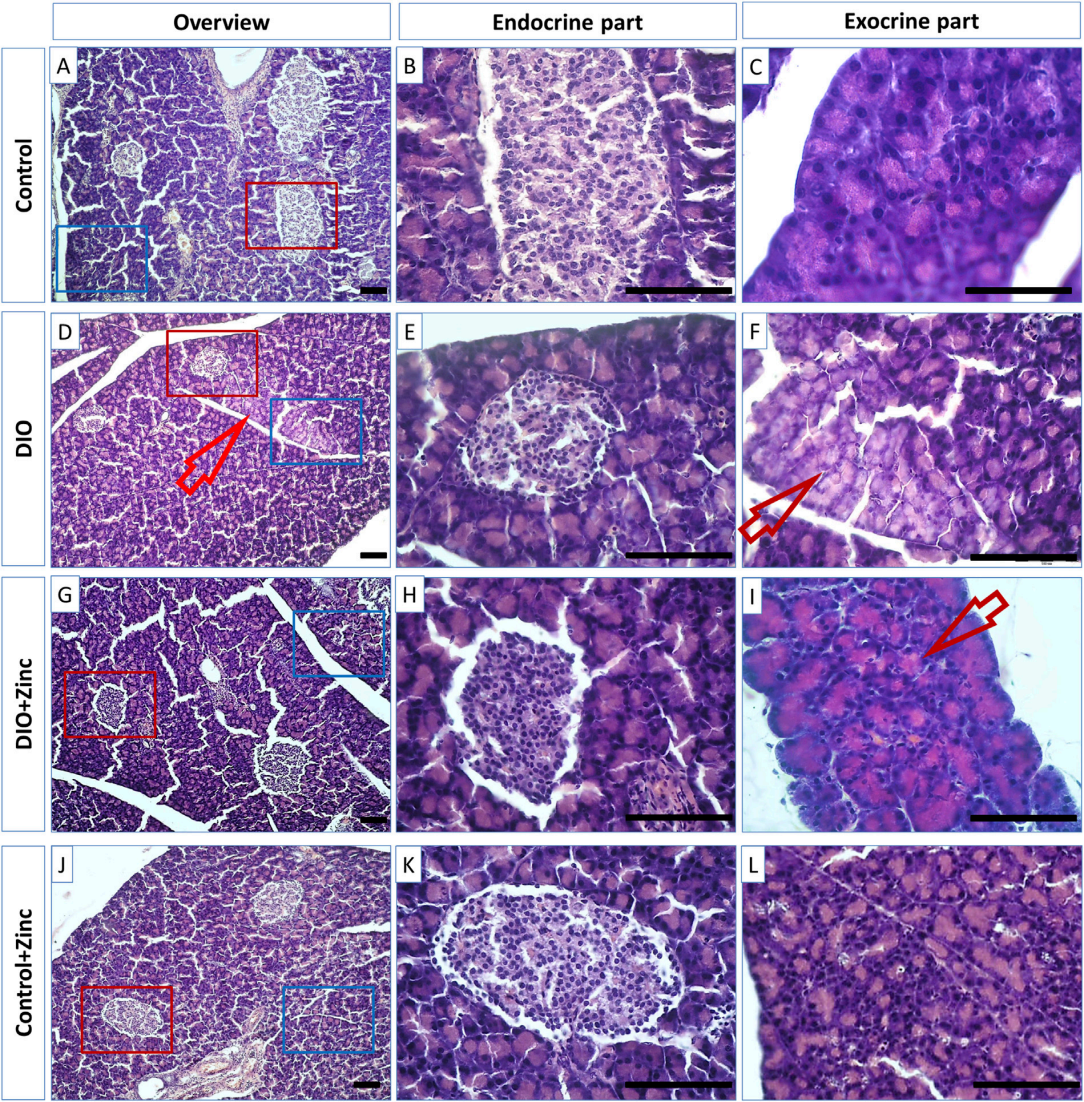
Microphotographs of the pancreas of rats from experimental groups: control **(A–C)**, DIO - Diet-induced obesity group **(D–F)**, DIO + Zinc - Diet-induced obesity group treated with Zn-64 stable isotope in aspartate form **(G–I)**, Control + Zinc - Control group treated with Zn-64 stable isotope in aspartate form **(J–L)**. **(B,E,H,K)** endocrine part of pancreas; **(C, F, I, L)**–exocrine part of pancreas. Hematoxylin and eosin staining. Arrow - lipid dysthrophic accumulations, scale bar 100 μm.

The pancreas functions as a mixed gland with both exocrine and endocrine components. The majority of the pancreas is composed of exocrine cells organized into acini, which secrete digestive enzymes. These secretions are transported out of the pancreas through a network of intercalated, intralobular, and interlobular ducts, eventually draining into the main pancreatic duct. In the control group (Figure 4), the acini exhibited a typical structure, characterized by granular and brightly acidophilic cytoplasm at the apical pole, and strongly basophilic nuclei at the basal pole.

In contrast, animal models of diet-induced obesity (DIO) (Figure 4) displayed acini with less pronounced eosinophilic apical cytoplasm (Figure 4, arrows), a condition likely resulting from lipid accumulation indicative of pancreatic fatty degeneration. However, the administration of ^64^Zn- aspartate to the rats maintaining standard diet did not alter the morphology of exocrine cells (Figure 4). Notably, the obese rats treated with ^64^Zn- aspartate (Figure 4) exhibited no evidence of fatty degeneration in the pancreatic tissue.

**FIGURE 5:**
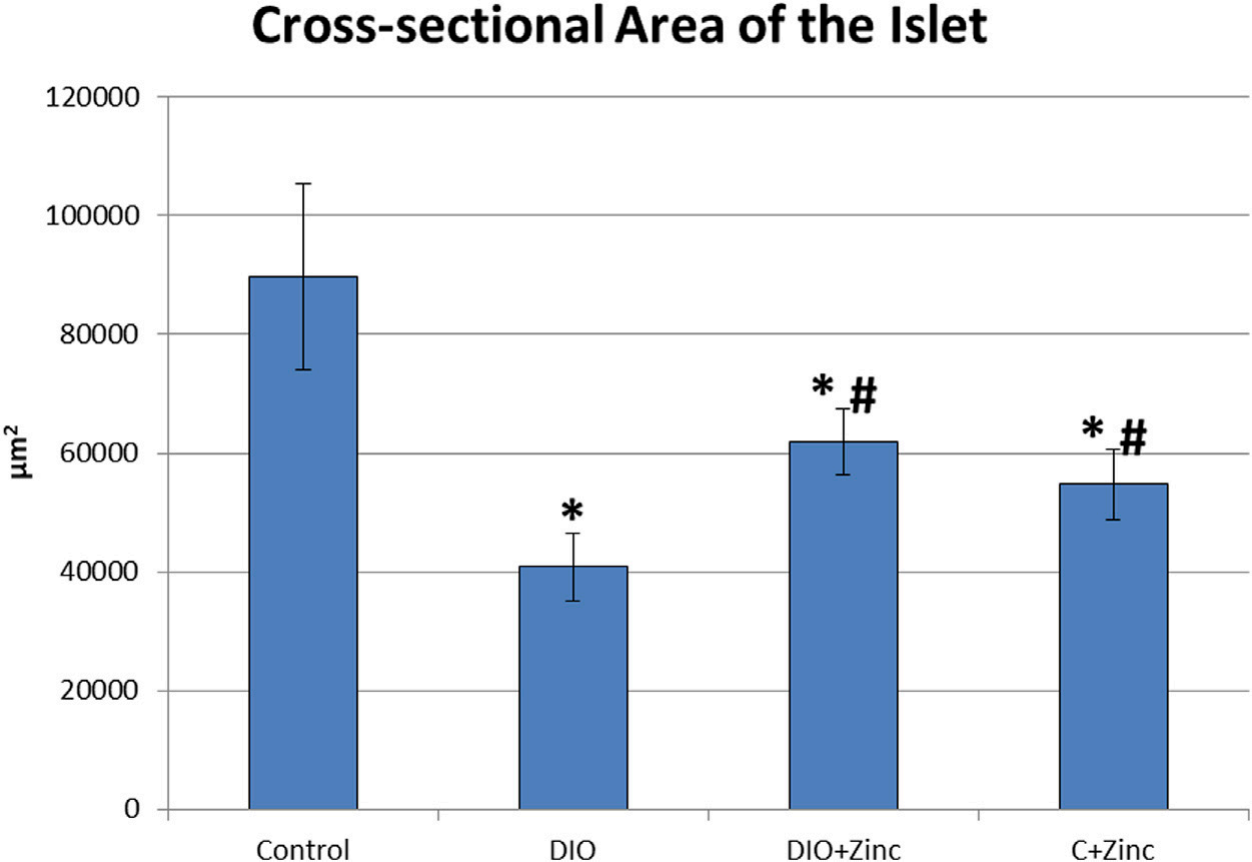
Cross-sectional area of the pancreatic islets. Data are presented as the mean value ± SEM. * - the difference is significant compared to the control group of animals; # - the difference is significant compared to the group of animal models of obesity.

The endocrine component of the pancreas consists of diffusely located islets. A morphometric analysis of the functional state of the endocrine part of the pancreas during the development of induced obesity revealed significant differences among the experimental groups (Figure 5). In the obesity group, the cross-sectional surface area of the islets was markedly reduced by 60%, indicating a substantial decline in the functional activity of the endocrine pancreas. However, in the obese rats treated with ^64^Zn- aspartate, the cross-sectional surface area of the islets increased by 43% compared to the obesity group, although it remained 29% lower than in the control group. Administration of ^64^Zn- aspartate to the rats on a standard diet resulted in a noticeable reduction in the cross-sectional surface area of the islets by 39% as compared to the control group.

There exists a direct relationship between the morphological and functional indicators of pancreatic health. The data obtained indicates that the hormone-synthesizing activity of the pancreas in rat models of diet-induced obesity was significantly diminished; however, this activity markedly increased with the administration of ^64^Zn- aspartate, although it did not fully return to the levels observed in the control group. Based on the observed improvement in islet cross-sectional surface area, it is plausible that a longer or continuous treatment with ^64^Zn- aspartate could further support islet repair and hormone-synthesizing activity in obese rats. Future studies with extended treatment durations could provide insights into the potential for enhanced regenerative or protective effects on islet architecture. Furthermore, there is evidence of improvement in the exocrine component of the pancreas following the administration of the test substance, as indicated by the disappearance of fatty degeneration, with no significant effects observed in the exocrine cells of rats maintained on a standard diet.

**FIGURE 6:**
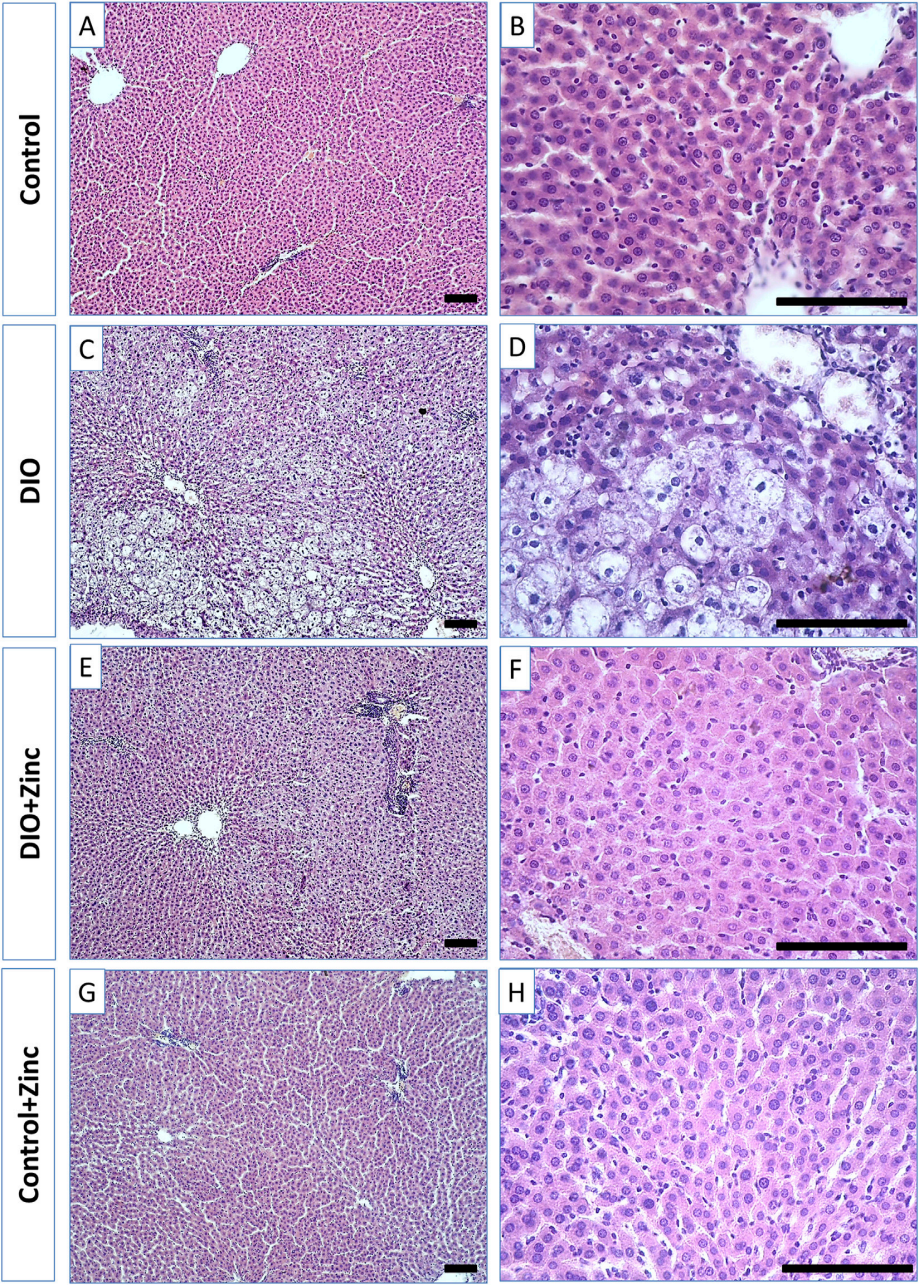
Microphotographs of the liver of rats from experimental groups: control **(A and B)**, DIO - Diet-induced obesity group **(C and D)**, DIO + Zinc - Diet-induced obesity group treated with Zn-64 stable isotope in aspartate form **(E and F)**, Control + Zinc - Control group treated with Zn-64 stable isotope in aspartate form **(G and H)**. Hematoxylin and eosin staining, scale bar 100 μm.

In control rats (Figure 6), the liver exhibited the classical lobular organization, characterized by a central vein running along the axis of each lobule. Hepatocytes, which are polygonal in shape with well-defined nuclei containing several nucleoli, are arranged into ordered hepatic cords radiating from the central vein. Binucleate hepatocytes are also present.

In the obesity group (Figure 6), the shape of the hepatocytes transformed from polygonal to rounded due to lipid inclusion deposition, indicative of fatty degeneration of the liver. Additionally, the structure of the hepatic cords becomes disarranged, and the number of binucleate cells in the field of view decreases. Administration of ^64^Zn-aspartate to the obese rats (Figure 6) restored the structure of the hepatic cords, with most hepatocytes regaining a polygonal morphology and showing no signs of fatty degeneration; binucleate cells were frequently observed. However, the trabecular disorganization remained. The administration of ^64^Zn-aspartate to rats on a standard diet (Figure 6) did not result in any changes in the morphology of the hepatocytes or the structure of the hepatic lobules.

**FIGURE 7:**
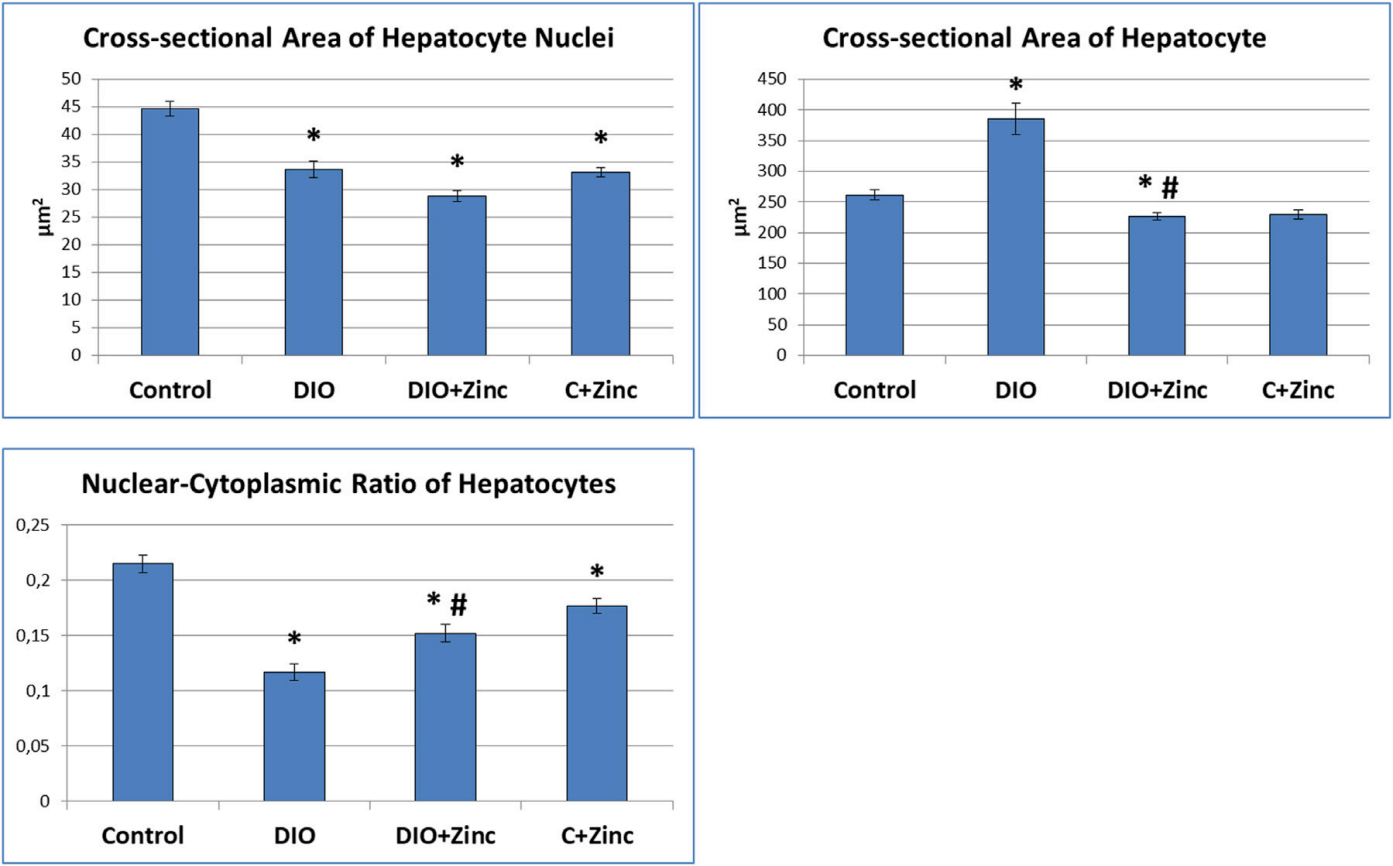
Morphometric analysis (сross-sectional area of hepatocyte nuclei, сross-sectional area of hepatocyte, nuclear-cytoplasmic ratio of hepatocytes) of the liver. Data are presented as the mean value ± SEM. * - the difference is significant compared to the control group of animals (p < 0.05); #- the difference is significant compared to the group of animal models of obesity (p < 0.05).

Significant morphometric changes occurred in hepatocytes during the progression of diet-induced obesity (Figure 7). In the obesity group, the nuclear area decreased by 25%, indicating reduced transcriptional activity, which was further evidenced by the nucleus’s dark coloration and homogeneous structure, with no nucleoli visible. In contrast, the area of the hepatocytes increased by 48% due to substantial lipid inclusion deposition. Consequently, the nucleus-to-cytoplasm ratio decreased significantly (by 45%), reflecting diminished cellular functional activity. Treatment with ^64^Zn-aspartate improved the morphometric parameters of the obese rats. Specifically, the area of hepatocytes in the animals treated with ^64^Zn-aspartate decreased by 41% compared to the untreated obesity models, which showed a 13% increase relative to control values, indicating reduced lipid accumulation in hepatocytes. Furthermore, the nucleus-to-cytoplasm ratio increased by 31% compared to the obesity group that showed a reduction of 30% as compared to control. Nevertheless, the nuclear area was reduced by 35% as compared to the control values, indicating a 14% decrease relative to the obesity group, which could be potentially due to the combined effects of a high-fat diet and ^64^Zn- aspartate on nuclear activity. In rats maintained on a standard diet, ^64^Zn- aspartate resulted in a reduction of the nucleus area by 26%, the area of hepatocytes by 12%, and the nucleus-to-cytoplasm ratio by 17%.

**FIGURE 8:**
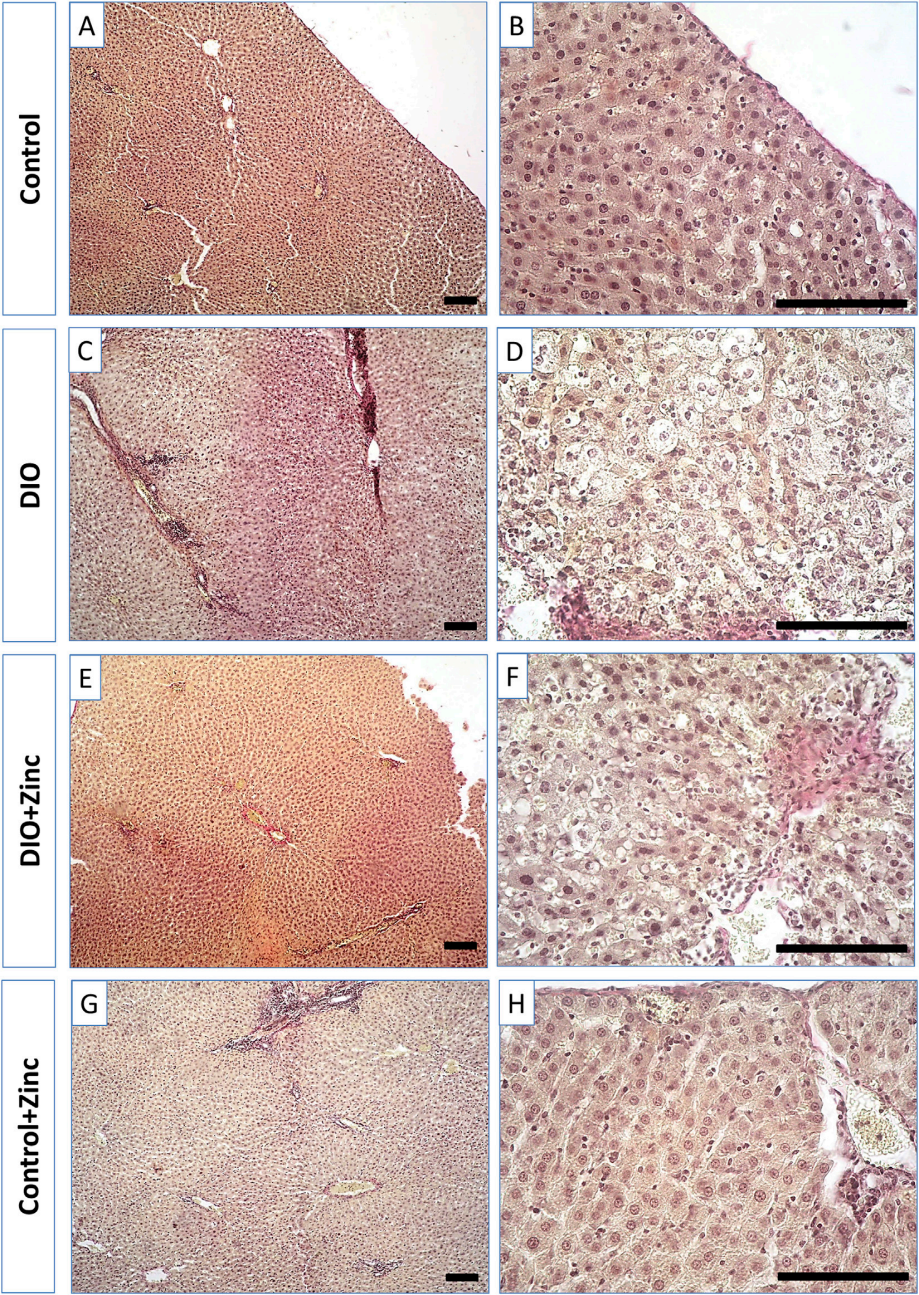
Microphotographs of the liver of rats from experimental groups: control **(A and B)**, DIO - Diet-induced obesity group **(C and D)**, DIO + Zinc - Diet-induced obesity group treated with Zn-64 stable isotope in aspartate form **(E and F)**, Control + Zinc - Control group treated with Zn-64 stable isotope in aspartate form **(G and H)**. Van Gieson’s staining method for the detection of collagen fibers (red color), scale bar 100 μm.

Liver fibrosis is characterized by excessive growth of connective tissue, along with increased synthesis and deposition of collagen in the extracellular matrix. In samples from the control group (Figure 8), most collagen fibers were located in the triads formed by small interlobular vessels.

Samples taken from the animals in the obesity group (Figure 8) demonstrated a marked increase in the number of collagen fibers within the triad region, which, as in the control group, consisted of small perilobular capillary plexuses and larger interlobular vessels. Similarly, samples from obese rats injected with ^64^Zn-aspartate form (Figure 8) revealed comparable levels of collagen fiber deposition in these areas when compared to those from the untreated obesity group. In contrast, the administration of ^64^Zn- aspartate form to rats on a standard diet (Figure 8) did not lead to significant changes in the quantity of collagen fibers in the perilobular and interlobular capillary plexuses.

**FIGURE 9:**
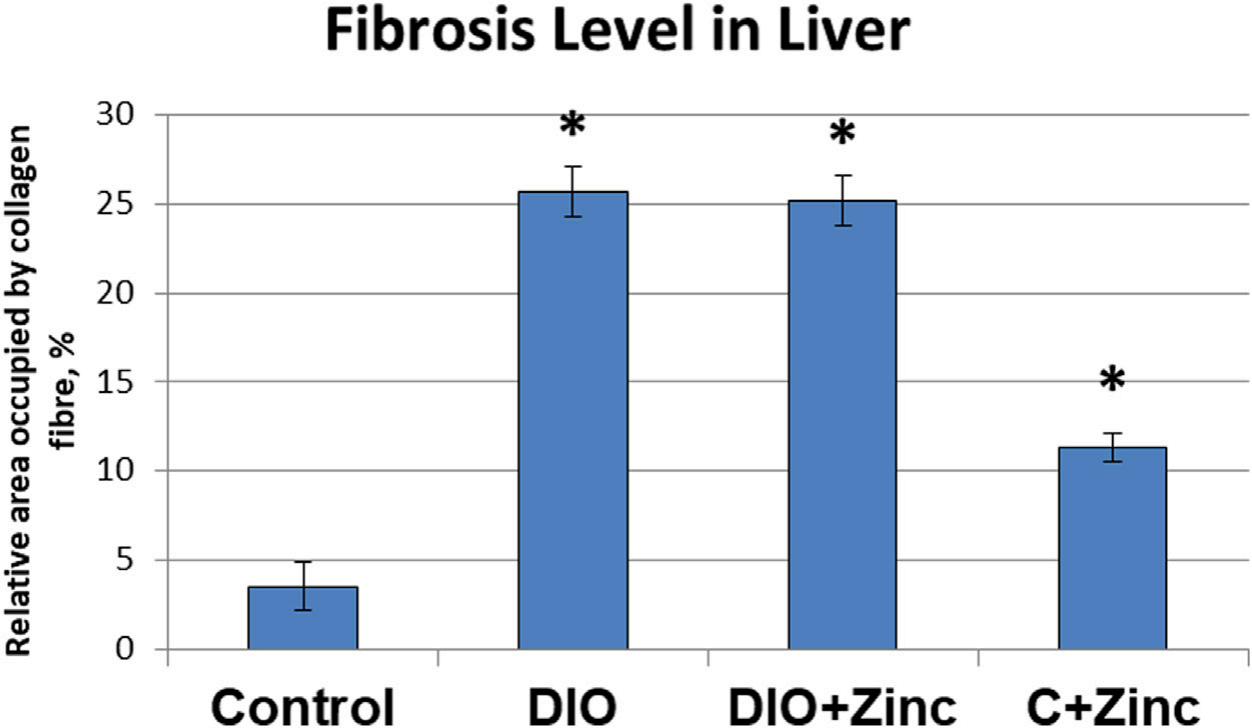
Fibrosis level in liver of rats from experimental groups. Data are presented as the mean value ± SEM. * - the difference is significant compared to the control group of animals; # - the difference is significant compared to the group of animal models of obesity.

An analysis of the area occupied by collagen fibers (Figure 9) revealed substantial changes associated with the development of induced obesity. In particular, the area of collagen fiber deposition in the obese group increased by 6.25 times as compared to the control. In the group treated with ^64^Zn-aspartate, the area of collagen fiber deposition increased by 6 times compared with the control group. No significant differences were observed between the untreated obesity group and the obesity group treated with ^64^Zn-aspartate. However, the administration of ^64^Zn-aspartate to rats on a standard diet resulted in a 2-fold increase in the area of collagen fiber deposition.

In summary, the findings suggest that ^64^Zn-aspartate exerts an improved histophysiology of the pancreas and liver in animal models of obesity.

### 3.3 Effects of ^64^Zn-aspartate on prooxidant-antioxidant balance in animal models of obesity

Given the importance of oxidative homeostasis, we assessed the impact of 64Zn-aspartate on prooxidant-antioxidant balance in obese animal models by measuring lipid peroxidation products—conjugated dienes (CD), TBARS, and Schiff bases (SB)—in serum. Obesity is known to induce systemic oxidative stress, leading to lipid membrane damage and the release of LOPs into circulation (Liu et al., 2022).

Our results showed a 1.86-fold increase in CD levels after 10 weeks of diet-induced obesity, indicating ongoing lipid peroxidation (Table 4). This likely reflects disrupted lipid metabolism and elevated plasma fatty acids, which serve as ROS substrates (Dludla et al., 2023). Correspondingly, TBARS levels were 4.8 times higher than controls, and Fe^2+^-ascorbate-induced TBARS formation exceeded control values by 20-fold, highlighting the role of non-enzymatic oxidation in worsening oxidative imbalance.

**TABLE 4:** Level of lipid peroxidation products in the blood serum of animals in the experimental groups (M ± SEM, n = 10).

	Conjugated dienes, nmol/mg protein	TBA-reactive substances, nmol/mg protein		Schiff bases, CU/mg protein
Experimental groups		Spontaneous accumulation	Fe^2+^-ascorbate-induced accumulation	
C	0.021 ± 0.001	0.006 ± 0.0003	0.033 ± 0.005	41.31 ± 2.47
DIO	0.039 ± 0.002^a^	0.029 ± 0.002^a^	0.61 ± 0.003^a^	168.86 ± 8.15^a^
DIO + zinc	0.025 ± 0.008	0.005 ± 0.0003^b^	0.15 ± 0.008^a^ ^,^ ^b^	56.27 ± 4.33^a^ ^,^ ^b^

Abbreviations: CU, conditional units; TBA, thiobarbituric acid.

^a^ The difference is significant compared to the control group of animals.

^b^ The difference is significant compared to the group of animal models of obesity.

Aldehyde LOPs, including TBARS, can form stable adducts with proteins, impairing their function and potentially triggering autoantibody formation (Zhou et al., 2020). Additionally, Schiff base levels were four times higher in obese animals, reflecting prolonged oxidative stress and accumulation of lipid peroxidation end-products.

These findings confirm that obesity induces chronic systemic oxidative stress, contributing to cellular damage and metabolic dysfunction. Moreover, elevated ROS levels may activate serine/threonine kinases (e.g., PKC, AKT, mTOR, GSK-3, p38 MAPK), promoting serine phosphorylation of IRS proteins and impairing insulin signaling, thereby facilitating insulin resistance (Barayeu et al., 2023).

Administration of ^64^Zn-aspartate form to animals resulted in the normalization of primary, secondary, and end LOP levels, further supporting the ability of ^64^Zn-aspartate form to modulate the overall prooxidant-antioxidant status of the body.

Our study demonstrated a significant increase in serum levels of oxidatively modified proteins in obese animals, with a marked elevation in aldehyde-dinitrophenyl-hydrazones, indicating active oxidative stress and associated metabolic dysfunction (Table 5). These elevations, alongside intensified lipid peroxidation, suggest sustained oxidative damage and activation of protein oxidation pathways, as evidenced by increased carbonyl derivatives with characteristic absorbance at 356 and 370 nm.

**TABLE 5:** Level of oxidative modification products of proteins in the blood serum of animals of experimental groups (M ± SEM. n = 10).

Groups	Aldehyde-dinitrophenyl-hydrazones. Nmol/mg protein	Ketone-dinitrophenyl-hydrazones. nmol/mg protein
C	0.187 ± 0.009	0.255 ± 0.023
DIO	0.698 ± 0.041^a^	0.571 ± 0.035^a^
DIO + zinc	0.253 ± 0.012^a^ ^,^ ^b^	0.200 ± 0.024^a^ ^,^ ^b^

^a^ The difference is significant compared to the control group of animals.

^b^ The difference is significant compared to the group of animal models of obesity.

While baseline levels of protein oxidation occur under physiological conditions due to normal protein turnover, the elevated carbonyl content in obese animals likely reflects both heightened ROS activity and impaired degradation of oxidized proteins (Gentile et al., 2017). This points to dysfunction in proteolytic regulation and enzyme activity under conditions of chronic oxidative stress.

Notably, 64Zn-aspartate treatment reduced levels of aldehyde-protein adducts compared to untreated obese animals, while ketone-protein derivatives remained comparable to control levels. These findings align with reduced lipid peroxidation and indicate a potential decrease in the intensity of free radical reactions.

In parallel, a significant decrease in superoxide dismutase (SOD) activity was observed in untreated obese animals, consistent with antioxidant system depletion. Treatment with 64Zn-aspartate led to a marked increase in SOD activity, exceeding both obese and control group values (Table 6), suggesting restoration of zinc-dependent enzymatic activity. This improvement may reflect normalization of zinc levels and reactivation of antioxidant defense mechanisms, particularly those involving zinc-dependent enzymes such as SOD.

**TABLE 6:** Superoxide dismutase and catalase activity in the blood serum of animals in the experimental groups (M ± SEM. n = 10).

	Experimental groups		
	C	DIO	DIO + zinc
Superoxide dismutase activity. CU/min per mg protein	3.36 ± 0.36	2.65 ± 0.41^a^	4.5 ± 0.43^a^ ^,^ ^b^
Catalase activity. µmol H_2_O_2_/min per mg protein	0.52 ± 0.05	0.43 ± 0.02^a^	0.48 ± 0.02^b^

Abbreviation: CU, conditional units.

^a^ The difference is significant compared to the control group of animals.

^b^ The difference is significant compared to the group of animal models of obesity.

These results support the role of 64Zn-aspartate in mitigating oxidative stress in obesity by reducing protein and lipid oxidation and enhancing endogenous antioxidant capacity.

## 4 Discussion

Obese individuals frequently exhibit hypozincemia (low serum zinc levels), which is inverse-correlated with oxidative stress markers. We did not measure the initial zinc levels in the animals of DIO group, acting upon an assumption that zinc levels were sufficiently reported to be significantly lower in the pancreas of obese mice, while higher in muscle, adipose tissue, and liver (Pei et al., 2022).

Zinc plays roles in the structure and catalytic activity of SOD1 and SOD3 enzymes. Our hypothesis is that isotopic enrichment of ^64^Zn could potentially influence the strength of coordination bonds between zinc and the protein’s amino acid side chains and rate of autocatalytic function.

Structurally, zinc serves as a scaffold within the Cu/Zn-SOD enzyme, helping to maintain its proper folding and conformation. The zinc ion is consistently bound to three histidine and one aspartate side chain, providing a stable coordination environment. Histidine coordinates zinc with its nitrogen atoms in the imidazole ring and aspartate does the same with oxygen atoms in its carboxyl side chain (Abi Habib et al., 2020). Both histidine and aspartate tend to preferentially bind isotopically heavier zinc, forming stronger bonds that require more cellular energy for disassociation. Zinc depletant conditions generally associated with obesity influence mitochondrial function, making less cellular energy available for Cu/Zn-SOD function.

Historically, the traditional assumption and understanding were that the impact of isotopic effects in biochemical processes was roughly proportional to the mass difference between isotopes. For example, the commonly accepted scientific stance has been that a 0.5% mass difference between standard enzymes and their ultralight counterparts (enzymes depleted in heavy isotopes such as ^13^C, ^2^H, ^15^N, and ^18^O) would result in a kinetic effect of about 1% or less. However, recent findings have revealed that this effect can actually be much more significant, ranging from 250% to 300%, depending on temperature (Franco and Canzoniero, 2024b).

This observed effect was found to be approximately 100 times greater than what was anticipated based on the traditional understanding. Another recent work shows that isotopic substitution can cause a fundamental change in the type of chemical bonds. Hence, the roles different stable isotopes may play in SOD and catalase activities and biochemical reactions were essentially ignored. In light of new findings, modern science must now update its hypotheses and develop new knowledgebase accounting for the significant influence of stable isotopes on physiobiological processes.

Frequently involved in Cu/Zn-SOD catalytic sites, histidine (His) binds zinc through its imidazole nitrogen, allowing for isotopic flexibility in coordination and facilitating enzymatic activity. This relative isotopic neutrality of histidine in metal binding could have important implications for zinc homeostasis and Cu/Zn-SOD function, as repleting intracellular zinc levels with isotopically light 64Zn may speed up catalytic functioning of superoxide dismutase by inducing antioxidant function of metallothioneins while inhibiting TNF-a signaling, while not significantly altering the isotopic composition of zinc pools in the body.

Metallothioneins (MTs) are small, cysteine-rich proteins that play critical roles in various biological processes, including protein synthesis within cells, primarily through their involvement in zinc homeostasis and regulation of cellular processes. Furthermore, metallothioneins, particularly MT-3, play a significant role in regulating autophagy and lysosomal functions, especially under conditions of oxidative stress, which is functionally important for Cu/Zn-SOD in the context of obesity. Metallothioneins bind zinc with cysteine residues, which contain thiol groups and preferentially bind isotopically light zinc by coordinating with its sulfur atoms. One of the primary functions of metallothioneins is to bind zinc ions with high affinity. This binding capacity allows MTs to act as intracellular reservoirs of zinc, storing the metal and releasing it as needed to maintain cellular zinc homeostasis (Chandran et al., 2010; Schilling et al., 2022) (Krężel and Maret, 2017; Mosna et al., 2023).

Under zinc-depleted conditions typically associated with obesity, proteins and amino acids would compete for the limited available zinc. Aspartate-containing binding sites may have an advantage in sequestering zinc due to their affinity. Hence, in zinc-depletant conditions, aspartate residues of SOD-1 and SOD-3 will bind isotopically light zinc, which tends to form longer and weaker bonds requiring less cellular energy for disassociation, hypothetically making more energy available to Cu/Zn-SOD function.

Isotopically light zinc (e.g., 64Zn) is generally more abundant and kinetically favored in biological systems. In zinc-depleted conditions, this preference for lighter isotopes may be more pronounced. We hypothesize that due to kinetic isotope effect, lighter isotopes (e.g., 64Zn) tend to form bonds faster due to lower reduced mass, which may induce wild type folding and function. This hypothesis requires further investigation.

Literature and data analysis on the involvement of ROS in protein oxidative degradation suggest that the reduction in SOD enzymatic activity may result from oxidative modification of the enzyme itself (Krężel and Maret, 2017). Since SOD is a metal-containing enzyme, ROS can directly damage the enzyme within its active site. In particular, hydroxyl radicals (OH¯), generated *via* Fenton and Haber-Weiss reactions from hydrogen peroxide and superoxide, act as direct agents that inactivate the enzyme (Mosna et al., 2023).

The rationale for using 64 Zn-Asp is based on studies showing that heavy zinc isotopes (e.g., ^68^Zn, ^70^Zn) are preferentially excreted in urine, while lighter isotopes are retained for essential metabolic functions (Ramana et al., 2017). This suggests that lighter zinc isotopes are more biologically active and efficiently incorporated into zinc-dependent enzymes and transporters.

Our study supports this hypothesis, as 64 Zn-Asp was more effective than natural zinc in improving metabolic markers such as BMI, glucose homeostasis, and lipid metabolism, because of Higher bioavailability (Lighter zinc isotopes are absorbed more efficiently in the intestine and transported more effectively into cells; **Enhanced enzymatic activity** [Enzymes such as SOD and catalase rely on zinc availability, and preferential uptake of ^64^Zn may facilitate faster enzyme activation (Chatgilialoglu, 2024)]; **Reduced toxicity risk** [Heavy zinc isotopes tend to accumulate in tissues, potentially leading to cytotoxic effects, whereas lighter isotopes are more efficiently utilized and cleared (Jaouen et al., 2016)].

The effects of zinc aspartate has extensively documented in various contexts. For instance, Barbarino et al. investigated the impact of zinc-aspartate and zinc-glycinate on gastric lesions in rats, demonstrating significant protective effects (Pasquini et al., 2022). Additionally, Yıldırım and Çıralık examined the effects of intraperitoneal administration of zinc aspartate on myringosclerosis in perforated rat tympanic membranes, providing further insights into its therapeutic potential (Moore et al., 2019b).

The reduction of oxidative stress markers by 64 Zn-Asp is a crucial component in mitigating obesity-related metabolic dysfunctions. Oxidative stress is well-documented as a key driver of obesity-associated complications, including insulin resistance, lipid dysregulation, and tissue fibrosis in metabolic organs such as the liver and pancreas (Barbarino et al., 1988).

In our study 64 Zn-Asp significantly reduced lipid peroxidation products (conjugated dienes, TBA-reactive substances, and Schiff bases) while increasing superoxide dismutase (SOD) and catalase activity. These changes are strongly associated with the reduction in systemic inflammation and improved insulin sensitivity. Notably, the reduction in oxidative stress markers correlated with improved glucose homeostasis, as evidenced by normalized serum insulin and glucose levels in the obese animals treated with 64 Zn-Asp (Table 3).

Previous studies have established that antioxidant interventions improve metabolic health by preventing oxidative damage to pancreatic β-cells, which are highly susceptible to oxidative stress-induced dysfunction (Yildirim et al., 2009). Our findings support this mechanism, as 64 Zn-Asp treatment preserved pancreatic islet morphology and function. Additionally, the observed improvement in lipid profiles (lower triglyceride and cholesterol levels) suggests that reducing oxidative stress contributes to better lipid metabolism, likely *via* the preservation of hepatic function and insulin sensitivity (Franco and Canzoniero, 2024).

In DIO models, pancreatic acinar cells often exhibit lipid accumulation, leading to pancreatic steatosis. This condition is characterized by increased oxidative stress within the pancreatic tissue. For instance, a study on Ossabaw swine fed a modified atherogenic diet reported significant pancreatic steatosis accompanied by elevated oxidative stress markers, such as increased pancreatic malondialdehyde levels (Banaszak et al., 2021).

Additionally, in Zucker diabetic fatty (ZDF) rats subjected to a chronic high-fat diet, fat accumulation within pancreatic acinar cells was observed, which appeared to be associated with subsequent pancreatic fibrosis (Severo et al., 2020).

Zinc supplementation has been shown to exert protective effects on pancreatic architecture. In streptozotocin-induced diabetic rats, zinc chloride treatment notably reduced oxidative stress and preserved pancreatic architecture, suggesting a protective role against diabetes-induced pancreatic damage (Fullenkamp et al., 2011).

Furthermore, zinc supplementation has been associated with improved glycemic control and modulation of pancreatic islet morphology, indicating its potential therapeutic role in managing obesity-related pancreatic alterations (Matsuda et al., 2014).

64Zn-Aspartate offers several unique advantages over traditional zinc supplements, including enhanced bioavailability (improving its intestinal uptake (Anton et al., 2022), the ability to track tissue-specific zinc distribution (Rech et al., 2024), and the potential to monitor the genetic regulation of zinc homeostasis *in vivo*. The use of the 64Zn isotope also provides an invaluable tool for personalized nutrition and precise metabolic interventions, making 64Zn-Aspartate a superior option for both clinical research and therapeutic applications in obesity, diabetes, and other metabolic diseases. Traditional zinc supplements, while beneficial in raising systemic zinc levels, do not provide the same level of detailed insight into zinc metabolism, tissue-specific effects, and gene expression regulation, underscoring the innovation and promise of 64Zn-Aspartate (Baron et al., 2016).

Several factors may have influenced the observed outcomes of DIO and zinc treatment: diet composition, physical activity, gut microbiota, baseline zinc status, genetic variability (Wang et al., 2019).

Obesity is linked to nutrient imbalances, dysbiosis, and compromised intestinal barrier integrity, which may be improved by polyphenols like curcumin and resveratrol through their effects on gut microbiota and zinc homeostasis. Zinc plays a key role in regulating enzymatic processes, maintaining microbial ecology, and preserving intestinal barrier integrity, all of which are crucial in mitigating obesity-related inflammation and enhancing gut health (Tian et al., 2020).

Obesity and diabetes are associated with reduced circulating zinc levels, which impair intracellular processes, particularly in the brain, where zinc plays a role in modulating synaptic activity and intracellular signaling. Altered expression of zinc-regulatory genes, such as ZNT and ZIP families, has been linked to obesity and may contribute to neurodegenerative diseases, as demonstrated by the reduced expression of ZNT1 and ZNT6 with increasing BMI, mirroring early-stage Alzheimer’s disease changes (Abdollahi et al., 2020; Islam et al., 2023; Olesen et al., 2016).

Future studies should consider these factors for a clearer interpretation of zinc’s role in obesity-related pancreatic changes.

### 4.1 Limitations

Several limitations exist in this study. First, the research was conducted solely in an animal model, limiting the direct applicability of the findings to human clinical practice. Additionally, the study design did not include a control group with a with natural zinc aspartate.

## 5 Conclusion

This study provides promising evidence for the potential use of 64Zn-aspartate in obesity management in animal models. However, given that these results are based on animal data, further research is needed to assess its safety and efficacy in humans. These findings offer a basis for future clinical investigations into its therapeutic potential.

1. It has been demonstrated that administration of ^64^Zn-aspartate to animals maintained on a high-fat diet resulted in a significant reduction in body mass index, weight, and food intake when compared to the untreated obese animal models.
2. ^64^Zn-aspartate has been observed to ameliorate histopathological changes in the pancreas and liver of animals subjected to a high-calorie diet, in contrast to untreated obese animal models.
3. The administration of ^64^Zn-aspartate form has been shown to normalize prooxidant-antioxidant homeostasis in animals fed a high-fat diet. This effect is achieved through a reduction in the intensity of free radical processes, evidenced by decreased levels of lipid peroxidation products and protein oxidative modification, alongside an enhancement of antioxidant defenses *via* increased activity of enzymes such as superoxide dismutase and catalase.

## Data Availability

The original contributions presented in the study are included in the article/supplementary material, further inquiries can be directed to the corresponding author.

## Abbreviations

KLS-1, ^64^Zn-aspartate; DIO, diet-induced obesity; TBA-active products, thiobarbituric acid-reactive substances; TCA, Trichloroacetic acid; DNPH, 2,4-dinitrophenylhydrazine; BMI, body mass index; LOPs, lipid peroxidation products; TBARSs, TBA-reactive substances; SB, Schiff bases; CD, conjugated dienes; SOD, superoxide dismutase.
